# Miocardite Aguda após a Vacina de mRNA contra a COVID-19

**DOI:** 10.36660/abc.20210469

**Published:** 2022-04-07

**Authors:** Daniel A. Gomes, Rita R. Santos, Pedro Freitas, Mariana S. Paiva, Jorge Ferreira, Marisa Trabulo

**Affiliations:** 1 Departamento de Cardiologia Hospital de Santa Cruz Centro Hospitalar de Lisboa Ocidental Carnaxide Portugal Departamento de Cardiologia, Hospital de Santa Cruz, Centro Hospitalar de Lisboa Ocidental, Carnaxide – Portugal

**Keywords:** Coronavirus-19, COVID-19, Vacina/efeitos Adversos, Imunogenicidade da Vacina, Mimetismo Molecular, Miocardite, Diagnóstico por Imagem

## Introdução

A vacinação é um dos maiores desenvolvimentos da medicina moderna e constitui um grande avanço na prevenção de doenças infecciosas.^1^ Normalmente as vacinas são eficazes e têm um excelente perfil de segurança geral.^2^De fato, relatos de efeitos adversos graves após a vacinação são extremamente raros e idiossincráticos.^2^

Como o mecanismo de ação da vacina é baseado na resposta imune do hospedeiro, uma estreita relação com a autoimunidade não pode ser desconsiderada.^3^ Casos de reatogenicidade imunológica, como síndrome de Guillain-Barré e miocardite aguda após vacinação, foram relatados anteriormente.^4 , 5^

Relatamos o caso de um paciente jovem do sexo masculino que desenvolveu miocardite aguda após a vacina de mRNA contra SARS-CoV-2.

## Relato de caso

Um homem de 32 anos foi hospitalizado com pré-síncope e dor torácica retroesternal opressiva. A dor perdurou por duas horas, não havia irradiação e não era alterada por movimentos respiratórios ou posição. Ele apresentava febre (39ºC) e mialgia generalizada por dois dias, iniciando um dia após a administração da segunda dose da vacina de mRNA contra a COVID-19. O paciente estava hemodinamicamente estável e seu exame físico na hospitalização foi normal, exceto pela presença de febre. Negou episódios recentes de dor torácica, infecção do trato respiratório ou gastrointestinal. Não fazia uso de drogas ou medicamentos e não foram identificados fatores de risco ocupacionais ou recreativos.

O paciente era saudável, exceto por um histórico de miopericardite idiopática, que ocorreu treze anos antes. Nessa ocasião, uma ressonância magnética cardíaca (RMC) realizada na fase aguda revelou realce tardio de gadolínio subepicárdico na parede lateral. O paciente recebeu alta e permaneceu estável, em acompanhamento clínico regular. A resolução completa desses achados foi observada na RMC realizada com 1 ano de seguimento.

**Figura 1:**
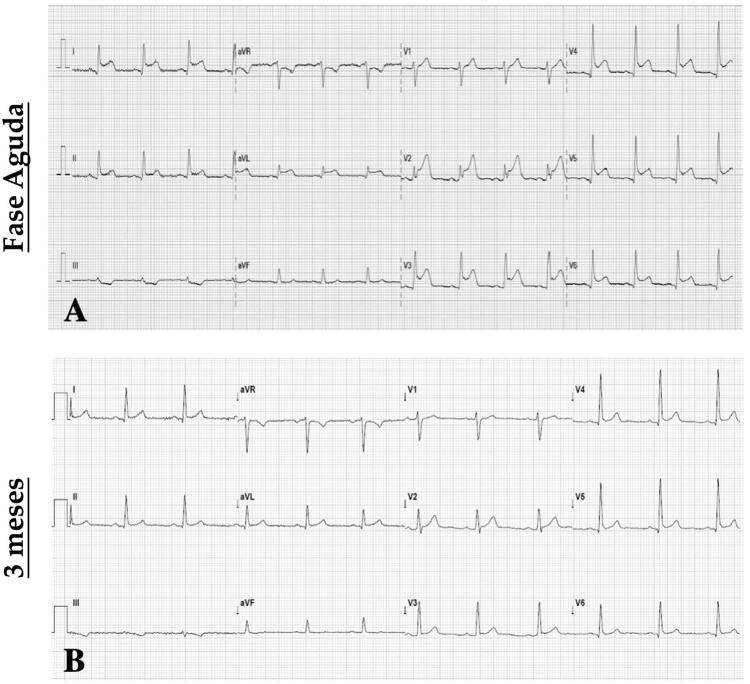
– ECG na hospitalização demonstrando supradesnivelamento difuso do segmento ST côncavo (figura 1A) e ECG aos 3 meses de seguimento mostrando resolução das anormalidades do segmento ST (figura 1B).

**Figura 2:**
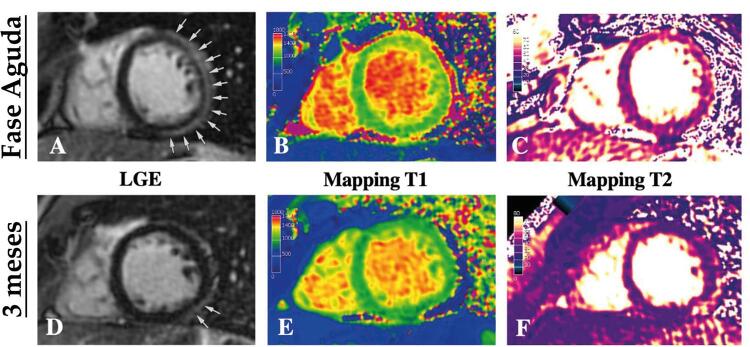
– Ressonância magnética cardíaca (RMC) na hospitalização demonstrando realce tardio de gadolínio subepicárdico nas paredes médio-anterior, lateral e inferior (figura 2A), e aumento de T1 (figura 2B) e T2 (figura 2C) nativos. A RMC aos 3 meses de seguimento revelou melhora no padrão subepicárdico de realce tardio de gadolínio (figura 2D) e normalização do T1 (figura 2E) e T2 (figura 2F) nativos.

Dadas as características da dor torácica em um paciente jovem com síndrome viral concomitante, a miocardite foi considerada como diagnóstico provável. O paciente apresentava parâmetros inflamatórios elevados (leucocitose e proteína C-reativa 4,6mg/dL) e biomarcadores miocárdicos (troponina cardíaca de alta sensibilidade T 834ng/L e NT-proBNP 433pg/mL) no exame de sangue. A radiografia de tórax foi normal. O ECG demonstrou supradesnivelamento difuso do segmento ST côncavo ( figura 1A ). Na ecocardiografia transtorácica, a fração de ejeção do ventrículo esquerdo estava preservada (58%) e não foram observadas anormalidades na contratilidade segmentar, embora o *strain* longitudinal global estivesse levemente reduzido (-17%). Não havia derrame pericárdico. A RMC revelou realce tardio subepicárdico nas paredes médio-anterior, lateral e inferior ( figura 2A ) acompanhado de aumento de T1 e T2 nativos nos segmentos médio-anterior e lateral ( figuras 2B e 2C ). Não foram observados sinais de inflamação pericárdica. A reação em cadeia da polimerase (PCR, *Polymerase Chain Reaction* ) de swab nasal e orofaríngeo foi negativa em duas ocasiões diferentes para SARS-CoV-2. Dada a alta suspeita clínica e um padrão de RMC consistente com miocardite aguda em um paciente sem fatores de risco cardiovascular conhecidos, a angiografia coronária não foi realizada.

O diagnóstico de miocardite aguda foi considerado. O paciente recebeu alta três dias após a hospitalização e foi aconselhado a não realizar atividade física intensa durante um período de 3 a 6 meses. Um ECG foi repetido após melhora clínica e revelou normalização do supradesnivelamento do segmento ST ( figura 1B ). Uma RMC aos 3 meses de seguimento demonstrou uma melhora significativa no padrão subepicárdico de realce tardio de gadolínio ( figura 2D ) e normalização das anormalidades anteriormente observadas no mapeamento das sequências T1 e T2 ( figuras 2E e 2F ).

## Discussão

A miocardite é uma doença inflamatória do miocárdio causada por várias condições infecciosas e não infecciosas.6 Sua apresentação clínica varia de forma ampla, de dor torácica leve até choque cardiogênico ou arritmias ventriculares potencialmente fatais.^6 , 7^ Embora a biópsia cardíaca continue sendo o padrão-ouro, não é rotineiramente realizada na prática clínica para a maioria dos pacientes e, portanto, a RMC, ao preencher os Critérios de Lake Louise modificados, é extremamente útil para estabelecer o diagnóstico.^6 - 8^

Há poucos relatos de miocardite após vacinação. Embora houvesse algumas preocupações iniciais sobre o desenvolvimento de doença cardíaca inflamatória em receptores de vacinas virais vivas, estudos mais recentes sugerem que seu risco geral não é aumentado.^5 , 9^ De fato, em uma coorte com mais de 41.000 pacientes, apenas um caso de pericardite definitiva e nenhum caso de miocardite foram diagnosticados nos primeiros 42 dias após a vacinação.^5^

A introdução de vacinas contra o SARS-CoV-2 é um elemento fundamental para controlar a propagação desta pandemia. Entre aqueles que receberam a vacina de mRNA contra a COVID-19 em ensaios clínicos de larga escala, ela mostrou-se altamente eficaz e segura, sem relatos de efeitos cardiovasculares adversos significativos.^10^ Sintomas sistêmicos relacionados à reatogenicidade imunológica foram comuns, principalmente leves a moderados, e mais frequentes após a segunda dose, com mediana de início de 1 a 2 dias após a aplicação da vacina.^11^

Relatamos o caso de um homem de 32 anos que desenvolveu miocardite aguda autolimitada após imunização contra a COVID-19. Este caso clínico é consistente com outros recentemente publicados.^12 - 14^ Como descrevemos, a miocardite aguda após a vacinação contra COVID-19 parece ser uma complicação potencialmente rara e autolimitada, afetando principalmente pacientes do sexo masculino jovens e saudáveis dois a três dias após receber a segunda dose.

Os mecanismos imunológicos exatos que ligam a vacina ao desenvolvimento de miocardite aguda não são completamente claros. Síndrome autoinflamatória, reatividade cruzada, mimetismo molecular e geração de autoanticorpos em indivíduos suscetíveis ou predispostos têm sido sugeridos como implicados na patogênese.^13^ De fato, relatos anteriores evidenciaram o papel da reação cruzada e do mimetismo nos fenômenos autoimunes pós-vacinação.^15^

Embora outras etiologias como miocardite viral coincidente com o momento vacinal não possam ser definitivamente excluídas, dada a associação temporal, podemos levantar a hipótese de que a resposta imune à vacina pode ter desencadeado a recorrência da miocardite neste paciente.

Mais estudos são necessários para esclarecer melhor a epidemiologia, a fisiopatologia e os resultados clínicos a longo prazo desses pacientes. Pesquisas futuras sobre esse assunto devem se concentrar em: (1) explorar fatores predisponentes e mecanismos fisiopatológicos para o desenvolvimento de lesão miocárdica após a vacinação contra COVID-19 (incluindo mimetismo molecular, formação de autoanticorpos e o papel de populações específicas de células imunes); (2) caracterizar alterações ultraestruturais e funcionais do miocárdio, bem como biomarcadores cardíacos e função cardíaca; (3) caracterizar prospectivamente a apresentação clínica, a evolução clínica e os resultados em longo prazo desses pacientes.

## Conclusões

A miocardite aguda autolimitada pode ser um efeito adverso potencial e raro das vacinas de mRNA contra a COVID-19. Embora os médicos devam estar cientes dessa possibilidade, de forma alguma devem desencorajar a vacinação, pois a análise de risco-benefício para a imunização contra a COVID-19 mostra um efeito benéfico consistente em todos os grupos.^14 , 16^ Atualmente a vacina é recomendada para todos com mais de 12 anos de idade.^16^
