# Supplementary material for: Miocardite Aguda após a Vacina de mRNA contra a COVID-19
Source: Arq Bras Cardiol. 2022 Apr 7;118(4):783–6. [Article in Portuguese] doi: 10.36660/abc.20210469 (PMC9007017; doi:10.36660/abc.20210469)
Supplement: Translation

# Translation: Acute Myocarditis Following mRNA COVID-19 Vaccine

**Authors:** Daniel A. Gomes, Rita R. Santos, Pedro Freitas, Mariana S. Paiva, Jorge Ferreira, Marisa Trabulo

**Affiliations:** 1 Portugal Departamento de Cardiologia, Hospital de Santa Cruz, Centro Hospitalar de Lisboa Ocidental, Carnaxide – Portugal

**Keywords:** Coronavirus-19, COVID-19, Vaccine/adverse effects, Immunogenicity Vaccine, Molecular Mimicry, Myocarditis, Diagnostic Imaging

## Introduction

Vaccination is one of the most important breakthroughs of modern medicine and constitute a major advance in the prevention of infectious diseases.^1^Overall, vaccines are effective and have an excellent general safety profile.^2^In fact, reports of severe adverse effects following vaccination are extremely rare and idiosyncratic.^2^

As the vaccine’s mechanism of action is based on the host immune response, a close relationship with autoimmunity cannot be disregarded.^3^Cases of immunologic reactogenicity, such as Guillain-Barré syndrome and acute myocarditis following vaccination have been previously reported.^4 , 5^

We report the case of a young male patient who developed acute myocarditis following the mRNA vaccine against SARS-CoV-2.

## Case report

A 32-year-old male individual was admitted with presyncope and oppressive retrosternal chest pain. The pain lasted for two hours, did not radiate and was not modified by respiratory movements or position. He had had fever (39ºC) and generalized myalgia for two days, starting one day after the administration of the second dose of the mRNA COVID-19 vaccine. The patient was hemodynamically stable and his physical examination at admission was unremarkable, except for the presence of fever. He denied any recent episodes of chest pain, respiratory tract or gastrointestinal infection. He was not taking any drugs or medications and no occupational or recreational risk factors were identified.

The patient was otherwise healthy except for a history of idiopathic myopericarditis, which occurred 13 years before. At that time, the cardiac magnetic resonance (CMR) assessment performed in the acute phase revealed subepicardial late gadolinium enhancement in the lateral wall. The patient was discharged and remained stable, under regular clinical follow-up. Complete resolution of these findings was observed in a CMR at one year of follow-up.

Given the characteristics of the chest pain in a young patient with a concomitant viral syndrome, myocarditis was considered as a likely diagnosis. The patient had elevated inflammatory parameters (leukocytosis and C-reactive protein 4.6mg/dL) and myocardial biomarkers (high-sensitive cardiac troponin T 834ng/L and NT-proBNP 433pg/mL) on blood analysis. The chest radiography was normal. The ECG showed diffuse concave ST-segment elevation ( figure 1A ). On transthoracic echocardiography, left ventricular ejection fraction was preserved (58%) and no abnormalities were observed in segmental contractility, although the global longitudinal strain was mildly reduced (-17%). There was no pericardial effusion. The CMR revealed subepicardial late gadolinium enhancement in the mid anterior, lateral and inferior walls ( figure 2A ) accompanied by increased native T1 and T2 in the mid anterior and lateral segments ( figures 2B and 2C ). No inflammation signs were observed in the pericardium. Naso- and oropharyngeal swab polymerase chain reaction (PCR) tests for SARS-CoV-2 were negative on two different occasions. Given the high clinical suspicion and a CMR pattern consistent with acute myocarditis in a patient without any known cardiovascular risk factors, coronary angiography was not performed.

**Figure 1:** – ECG at admission demonstrating diffuse concave ST-segment elevation (figure 1A) and ECG at three months of follow-up showing resolution of ST-segment abnormalities (figure 1B).

**Figure 2:** – Cardiac magnetic resonance (CMR) at admission demonstrating subepicardial late gadolinium enhancement (LGE) in the mid anterior, lateral and inferior walls (figure 2A), and increased native T1 (figure 2B) and T2 (figure 2C). The CMR at three months of follow-up revealed improvement in the subepicardial late gadolinium enhancement pattern (figure 2D), and normalization of native T1 (figure 2E) and T2 (figure 2F).

The diagnosis of acute myocarditis was assumed. The patient was discharged three days after admission and advised against intense physical activity during a three to six-month period. An ECG was repeated after clinical improvement and revealed normalization of the ST-segment elevation ( figure 1B ). A CMR assessment at three months of follow-up demonstrated significant improvement in the subepicardial late gadolinium enhance pattern ( figure 2D ) and normalization of the previously observed abnormalities in mapping T1 and T2 sequences ( figures 2E and 2F ).

## Discussion

Myocarditis is an inflammatory disease of the myocardium caused by a variety of infectious and non-infectious conditions.6 Its clinical presentation varies widely, ranging from mild chest pain to cardiogenic shock or life-threatening ventricular arrhythmias.^6 , 7^Although the cardiac biopsy remains the gold standard, it is not routinely performed in clinical practice for most patients and, therefore, the CMR, by meeting the modified Lake Louise Criteria, is extremely useful in establishing the diagnosis.^6 - 8^

There are only a few reports of myocarditis following vaccination. Although there were some initial concerns about the development of inflammatory cardiac disease in live viral vaccine recipients, more recent studies suggest its overall risk is not increased.^5 , 9^In fact, in a cohort including over 41000 patients, only one case of definitive pericarditis and none of myocarditis were diagnosed in the first 42 days after vaccination.^5^

The introduction of vaccines against SARS-CoV-2 is a key element in controlling the spread of this pandemic. Among those receiving mRNA COVID-19 vaccine in large-scale clinical trials, it proved to be highly effective and safe, with no reports of significant adverse cardiovascular effects.^10^Systemic symptoms related to immunologic reactogenicity were common, mostly mild to moderate ones, and more frequently observed following the second

dose, with a median onset of 1-2 days after vaccine application.^11^

We report a case of a 32-year-old man who developed self-limited acute myocarditis after COVID-19 immunization. This clinical case is in line with recently published ones.^12 - 14^As we described, acute myocarditis following COVID-19 vaccination appears to be a potentially rare and self-limited complication, affecting mostly young and healthy male patients two to three days after receiving the second dose.

The exact immunological mechanisms linking the vaccine to the development of acute myocarditis is not completely clear. Autoinflammatory syndrome, cross-reactivity, molecular mimicry, and autoantibody generation in susceptible or predisposed individuals have been suggested to be implicated in pathogenesis.^13^In fact, previous reports have elicited the role of cross-reaction and mimicry in post vaccination autoimmune phenomena.^15^

Although other etiologies such as viral myocarditis coincidental with the vaccination timing cannot be definitively excluded, given the temporal association, we can hypothesize that the immune response to the vaccine may have triggered the recurrence of myocarditis in this patient.

More studies are needed to further clarify the epidemiology, pathophysiology, and long-term clinical outcomes of these patients. Future research on this subject should focus on: (1) exploring predisposing factors and pathophysiological mechanisms for the development of myocardial injury following COVID-19 vaccination (including molecular mimicry, autoantibody formation, and the role of specific immune cell populations); (2) characterizing myocardial ultrastructural and functional changes, as well as cardiac biomarkers and cardiac function; (3) prospectively characterizing these patients’ clinical presentation, clinical course and long-term outcomes.

## Conclusions

Self-limited acute myocarditis may be a potential and rare adverse effect of mRNA COVID-19 vaccines. While clinicians must be aware of this possibility, by no means it should discourage vaccination, as the risk-benefit analysis regarding COVID-19 immunization shows a consistent beneficial effect across all groups.^14 , 16^The vaccine is currently recommended for everyone aged ≥ 12 years.^16^
